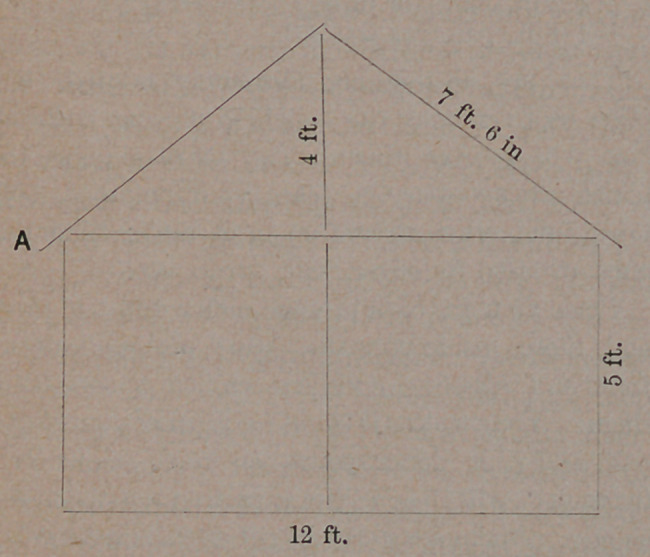# Summering

**Published:** 1878-07

**Authors:** 


### SUMMERING.

It has not only become fashionable or customary, but in many instances necessary, for people actively employed, to seek recreation or rest away from home, or from the scene of their labors, during the summer months.

The places selected are as various and the surroundings as different, as are the habits or inclinations of the people who seek them.

The wealthy matron, with her bevy of marriageable daughters, seeks Saratoga, Cape May, or some equally fashionable and expensive resort, where the time is spent in dressing, eating rich and indigestible food, and taxing the nervous system with dancing and late hours of dissipation. This constitutes her rest. Others, with less means or inclination, visit the summer boarding houses that dot the banks of almost every lake, where a bass, perch or pickerel may be captured, as an inducement for "sport." High mountains, wonderful glens, charming rivers and quiet villages, all offer their inducements to the seeker after a place to summer, and all are attended with more expense than many of us can afford.

We want to tell you how to spend the hot months in the most delightful manner, and at less than one-quarter the cost that you are subjected to at any of the places first noted. In order that our instruction may be practical and therefore available, we will enter into details.

Our plan involves camping out in tents that are tight and water-proof, yet cool and airy.

Do not buy your tents, they are far too costly; make them. We will tell you how:—Mark out a diagram on your barn floor like this, and we will show you how to construct a tent 12x18 feet, that shall be water-tight, and cost you less than ten dollars.

From the diagram, it will be seen that you will require 6 strips of "duck," 7 feet and 6 inches long, making in all 15 yards. These must be sewed in a double seam, overlapping each breadth one inch. Make a hem one inch wide, upon the two ends that form the eaves. Through this run a rope the size of an ordinary bed cord; at every breadth work an eyelet to receive the cords that hold the tent to the pegs. The cord running through the hem prevents the material from tearing. The duck should not cost more than sixteen cents per yard, making the roof to the tent cost $2.40. You will now require for the two ends, 21⅓ yards of heavy unbleached muslin, worth eight cents a yard, costing therefore, $1.71. Cut this into four strips 7 feet long, and four more 9 feet in length. Lay the two long ones in the centre and the two short ones on each side of them, over your diagram on the floor, and cut off the corners so as to have them fit into the gable of the roof. Sew them together, except in the centre, when one end of the tent is formed. Serve the remaining four pieces in like manner for the opposite end, then sew them to the roof.

You will next need twelve strips of the same material, 5 feet long, for the sides. Sew six of them together for each side, overlapping the seams one inch, as in the top, and hemming it one inch wide at the bottom. Pass a rope through this hem, and work eyelets at every seam. Sew the sides thus formed, to the top, so as to allow it to project three inches, as seen at A in our diagram.

This requires 20 yards of goods, costing $1.60. Now, sew your corners together, and work large button holes upon one of the centre end pieces, from the ground to the eves; on the opposite one, sew large wooden buttons, so that the ends can be buttoned together in case of a storm. Next, you will need a fly. For this you will require six pieces of muslin 10 feet long, which, when sewed together and hemmed in the same manner as the top, with rope and eyelets in same localities, will form a shelter over the entire tent, projecting fifteen inches beyond, to carry the water free from the sides. This fly catches the first shock of the rain and prevents it falling through the real roof of the tent in a fine spray. It also keeps your tent cooler when the sun falls upon it. For this, twenty yards of muslin is necessary, costing $1.60.

You now have

| | |
|---|---|
| 15 yds. of duck @ 16c. | $2.40 |
| 61⅓ yds. muslin @ 8c. | 4.91 |
| Necessary rope, say. | 2.00 |
| | $9.31 |

making your tent complete, costing you less than ten dollars, not estimating your labor at anything.

When searching for a tent of this capacity, we were asked eighty dollars for it by a manufacturer, and of no better material than the one we made ourselves. Seven cords ten feet long, will be required, with one end tied in the eyelet made at every seam along the eves. Marlin or tarred rope, about a foot long, must be tied in the eyelets at the bottom of the tent, to hold it to pegs which will be driven in the ground to hold the sides perpendicularly. Your fly will also need four ropes on each side, about fifteen feet long.

Now, make a strong brine, using all the salt the water will dissolve, and plunge your tent and fly into the pickle, where it may remain two or three days. At the end of that time remove it, and spread it on a line to dry. Then go over it with a very thin white-wash, made from fresh lime, putting the solution on with a white-wash brush. When dry, your tent is ready for use, will never mildew or rot, and will be ready for service during your natural life. When you take it to the woods, a ridge pole eighteen feet long can be cut, together with two upright poles nine feet high with a fork upon the small ends to receive and hold it in place. The pins and stakes can be cut in the woods in like manner, so saving trouble of transportation.

If you require a tent for your servant, sew three pieces of ducking together, fifteen feet long. Hem and work eyelets across the ends. Mark out your diagram on the floor as for the large tent, only making this one in shape of the letter A. Estimating your ridge pole to be 6½ feet high, and the sides of your tent reaching the ground, you will require two strips for each end 6½ feet long, with corners cut to fit your gables. These, when sewed in the ends, and left open in the middle, at one end of the tent, will give you an A tent 6x9 feet on the ground, and costing less than $3.50.

As it is certain to rain more in the woods than when you are at home, you had better prepare another "fly," made of striped awning cloth, of about the size of the fly to your tent, and made in the same manner. This will serve you as a dining room when stretched over a ridge pole, supported by two forked poles planted in the ground, to hold them firmly. These poles should be ten feet high, and the eves of your roof should not come nearer than five feet of the ground. Your table, made of drift wood, always to be found on a stream, or of branches of trees of about an inch in diameter, laid close together, and nailed upon suitable cleats, supported by four legs driven into the ground, should be placed in the centre of this canopy. If you do not take camp stools, make them by boring four holes in pieces of slabs, or blocks of wood that are always at hand on all habitable streams of this day, and drive legs into them. For this and other camp purposes, you will need a saw, 1½ inch auger, hatchet, a four pound axe, and a quantity of nails. With these implements, you can construct a home and furnish it comfortably and even elegantly, in two or three days, and enjoy every moment of the time you are so employed.

Now, who's going? If you take your wife and children—which we recommend, as they can be perfectly comfortable in such a tent, and will grow strong and happy in the open air; we advise you to locate on the bank of some stream, in a shady wood, near some farm house that can supply you with bread, butter, milk and eggs.

If a party of four gentlemen are going to spend four weeks in trout or other fishing, select a dry spot under some large trees, near the best fishing pools, and take with you the following necessary utensils:

Two long handled frying pans.
A coffee and tea pot.
A long handled 8 qt. boiler [in which to boil your potatoes and to heat your dish water.]
A dish pan.
A small tea kettle.
A wire broiler.
Six common knives and forks.
1 butcher knife.
1 long handled iron spoon.
1 " " " fork.
1 dozen tin teaspoons.
½ " " table spoons.
12 tin plates.
12 " cups.
1 2 qt. pail [for milk.]
1 4 qt. pail [for water.]

These are all that are absolutely needed, but other useful articles can be added if your packing box will receive them. The extra number of plates are necessary to place your potatoes, fish, bread, and other articles upon, at table, while the cups are used for sugar, salt, syrup, &c.

Place your tin cups in your coffee and tea pots, and these inside your dish pan; putting knives, forks and spoons in the unoccupied space about them. Your teakettle should go inside your 4 qt. pail, and your dozen plates on top. Your own ingenuity will teach you how to pack them all snugly. Make a strong box of inch pine boards, four feet long, two feet wide, and two feet deep. Bind the corners with iron, and strengthen the top in same manner. Attach the top to the box by means of long strap hinges, and a strong hasp in front to hold it down. Eight inches from the top, at each end, bore two one-inch holes, through which put a piece of inch rope, wrapping the ends together on the inside, so as to form handles by which to lift it. In the bottom of this box pack the following provisions, which will keep four hungry persons four weeks, as well as any visitors that are likely to call upon you and enjoy your hospitality:

12 lbs. wheat flour.
20 " corn meal.
4 quarts beans.
2 bushels potatoes.
1 peck Bermuda onions.
5 lbs. rice.
10 " dried fruit, [peaches are best.]
10 " ground coffee.
5 " black tea.
25 " "A" sugar.
10 " salt pork, [in which to fry fish.]
10 " dried beef.
12 " butter, [in crock.]
½ " black pepper.
⅛ " red pepper.
2 " candles.
5 " soda crackers.
2 qts. pickles.
1 sack salt.
1 package matches, [tightly corked in bottle.]
1 box baking powder.
1 " saleratus.
Soap and towels.

To this add a quantity of bread and biscuit, and rely upon some farm house for a fresh supply.

Your sugar should be placed in a sugar bucket with cover, to be found at all grocers. Your coffee and tea may go into tin cans, as well as your flour and corn meal. The tin cans used for fancy crackers, to be had of your grocer, will be very convenient for packing such articles, and will fit nicely in your camp chest. After your provisions are in the box, throw your potatoes and onions in among them, to fill up the vacant spaces, then pack your cooking utensils, with axe, saw, hatchet, auger and nails, on top, together with a bar of iron a half inch thick, an inch and a half wide, and as long as the box. Your box will contain them all and your tent to boot!

You can add to this list canned fruits, tomatoes, corn, &c., with some jellies and jam, that taste very well in the woods. We have only given a list of what seems to be necessary. If you smoke, do not forget your pipes and Vanity Fair tobacco. Do not take cigars. They soon become damp and do not burn well.

Erect upon one side of your tent two bedsteads, constructed in the following manner: Drive six green stakes solidly into the ground, forming two rectangles, end to end, four by six feet. Cut two straight saplings, twelve feet long, and nail them to the three stakes on a side, about eighteen inches from the ground. Across these nail slats, formed of round poles of as near the same size as possible, and across the ends other poles, to serve as "head boards." This gives you two bedsteads, foot to foot, upon which you can place two ticks, of suitable size, filled with straw from the nearest farm house. If you are away from civilization, where straw or hay cannot be obtained, gather a quantity of hemlock or spruce boughs, pile them up a foot or more deep, over which spread a blanket or comfortable, and you will have a bed fit for a king to rest upon. In a large trunk or box you can pack six comfortables, four sheets and four pillows, all the bed clothing required, even should the weather be cold. A comfortable placed over a straw tick, makes a luxurious bed. Once in two or three days, the beds and bedding should be spread in the sun, else are they apt to grow musty.

Near your dining room, pile up a lot of stones, about a foot high and four feet long. Build a wing on each end two feet long, cover the stones with sods and earth, (and this reminds us that you will need a shovel) so that when it is finished you will have a fire-place shaped like this: [‾‾‾‾]

Now, place your four feet bar of iron across the top of this, upon which to set your frying pans, your coffee and tea pots, and your boiler, making one of the most convenient arrangements for outdoor cooking that can be devised. Under this bar of iron, protected by the banks of stones and sods to the rear and on either side, hot glowing coals are always kept, and over which fried fish, flap-jacks, boiled potatoes, coffee and other food can be conveniently cooked.

If your neighboring farm house has ice, sink a flour barrel in the ground to its top, boring a few holes in the bottom for drainage, and place a large chunk of ice in the bottom, covering the top with a board or blanket. If a barrel is not to be had, dig a hole two or three feet deep and stone it up, where you can keep your milk, butter and fish cool and fresh, even without ice.

You are now ready for house-keeping, and if you cannot devise means for enjoying yourselves in the woods, and upon a fishing stream or lake, during the hot months now coming, we are sorry for you.

We could tell you how we busied ourselves every June for eight years past, but our space will not permit, and our article we fear is too long already. At some other time we may continue our narrative. If we have shown you how you may camp out comfortably, healthfully and economically, and can induce you to try the experiment just once, we have no fear but that we will find you enjoying yourself in the glorious woods every summer thereafter.

## Figures and Tables

**Figure f1:**